# Total scalp irradiation: A study comparing multiple types of bolus and VMAT optimization techniques

**DOI:** 10.1002/acm2.14260

**Published:** 2024-01-19

**Authors:** Tanisha M. Davis, Kirk Luca, Lisa J. Sudmeier, Zachary S. Buchwald, Mohammad K. Khan, Xiaofeng Yang, Eduard Schreibmann, Jiahan Zhang, Justin Roper

**Affiliations:** ^1^ Medical Dosimetry Program Southern Illinois University Carbondale Illinois USA; ^2^ Department of Radiation Oncology Emory University Atlanta Georgia USA

**Keywords:** air gaps, bolus, gEUD, optimization, total scalp, VMAT

## Abstract

**Purpose:**

To investigate bolus design and VMAT optimization settings for total scalp irradiation.

**Methods:**

Three silicone bolus designs (flat, hat, and custom) from .decimal were evaluated for adherence to five anthropomorphic head phantoms. Flat bolus was cut from a silicone sheet. Generic hat bolus resembles an elongated swim cap while custom bolus is manufactured by injecting silicone into a 3D printed mold. Bolus placement time was recorded. Air gaps between bolus and scalp were quantified on CT images. The dosimetric effect of air gaps on target coverage was evaluated in a treatment planning study where the scalp was planned to 60 Gy in 30 fractions. A noncoplanar VMAT technique based on gEUD penalties was investigated that explored the full range of gEUD alpha values to determine which settings achieve sufficient target coverage while minimizing brain dose. ANOVA and the *t*‐test were used to evaluate statistically significant differences (threshold = 0.05).

**Results:**

The flat bolus took 32 ± 5.9 min to construct and place, which was significantly longer (*p* < 0.001) compared with 0.67 ± 0.2 min for the generic hat bolus or 0.53 ± 0.10 min for the custom bolus. The air gap volumes were 38 ± 9.3 cc, 32 ± 14 cc, and 17 ± 7.0 cc for the flat, hat, and custom boluses, respectively. While the air gap differences between the flat and custom boluses were significant (*p* = 0.011), there were no significant dosimetric differences in PTV coverage at V57Gy or V60Gy. In the VMAT optimization study, a gEUD alpha of 2 was found to minimize the mean brain dose.

**Conclusions:**

Two challenging aspects of total scalp irradiation were investigated: bolus design and plan optimization. Results from this study show opportunities to shorten bolus fabrication time during simulation and create high quality treatment plans using a straightforward VMAT template with simple optimization settings.

## 1 INTRODUCTION

Total scalp irradiation (TSI) is indicated for angiosarcoma and advanced skin cancers of the scalp.^1^, ^2^ TSI presents technical and dosimetric complexities due to the shallow depth of the concave target, which surrounds the uninvolved brain. Because the scalp is in the photon buildup region, a tissue equivalent bolus is often placed over the scalp to increase the superficial dose. The custom fabrication of a patient‐specific bolus is a time‐consuming process, leading to long simulation sessions that can create a workflow bottleneck in the clinic, yet it is imperative to carefully craft the bolus for comfort and reducibility. Moreover, the bolus construction is specifically tailored for adherence to the scalp thereby minimizing air gaps, which could potentially lead to underdosing of the scalp. Another complexity of treatment planning arises from the necessity to arrange photon beamlets in such a way that they predominately irradiate the scalp tangentially while minimizing the path length into the healthy brain tissue or other radiosensitive organs at risk (OARs) such as the hippocampal substructure, lacrimal glands, lenses, and uninvolved skin.^3^, ^4^, ^5^, ^6^, ^7^ Due to the intricate nature of these challenges and the infrequent occurrence of TSI treatments, notable variations arise in both bolus fabrication techniques and the quality of treatment plans in clinical practice.

Several treatment techniques have been investigated for TSI. A classic technique for TSI uses two opposed lateral enface electron fields to treat the central regions with two opposed lateral photon fields dosing the peripheral rind. A significant challenge to this approach is matching the electron and photon fields to ensure accurate dose in the junction.^8^ In the intensity modulated radiation therapy (IMRT) era, static field and arc‐based photon plans have been explored for TSI.^9^ An advantage of IMRT or volumetric modulated arc therapy (VMAT) is eliminating the electron‐photon junction. However, IMRT and VMAT plan quality depends on beam geometry and the optimization settings. For an individual case, the mean brain dose varied by up to 10% across coplanar IMRT, noncoplanar IMRT, and VMAT arrangements.^9^ Further, in a case study where VMAT was used to treat the total scalp to 60 Gy, the lower isodose levels covered much of the healthy brain: V5Gy (100%), V10Gy (75%), and V20Gy (35%).^10^ Given the link between brain dose and cognitive decline,^3^, ^4^, ^5^ brain sparing is an important consideration in TSI.

Numerous radiotherapy clinics have investigated various bolus designs for TSI over the past several years to help obtain a more homogeneous dose to the total scalp.^11^, ^12^ While Superflab is commonly cut and pieced together to form a helmet, it can be cumbersome to craft and may not be reproducible for daily treatments.^13^ Thermoplastic sheets are another commercial option that when heated, soften and easily conform to the scalp, helping to reduce air gaps. However, once the plastic bolus dries, it will harden and can become uncomfortable, even painful, increasing the chance of setup error. There are also uncertainties with thermoplastic bolus thickness during the simulation process. The bolus is often stretched to achieve conformity and coverage of the designated treatment area resulting in a non‐uniform bolus thickness. While there are various commercially available boluses, it can be challenging with these to gain complete contact with the uneven surface of the patient's skin.^14^

There are broad applications of three‐dimensional (3D) printed treatment aids in radiotherapy, including patient‐specific custom bolus.^15^, ^16^ Custom bolus using 3D‐printed molds offers the potential to reduce air gaps, increase clinical efficiency, and help achieve uniform dose to the scalp.^17^, ^18^ An investigation of this technique where liquid polyurethane resin was poured into a 3D‐printed mold produced a patient‐specific custom bolus with enhanced target homogeneity in comparison to commercial bolus.^19^ While these investigations are encouraging, custom bolus inherently adds complexity and effort to the simulation. Another approach is to leverage 3D printing technology to prefabricate generic scalp boluses based on representative head sizes, which could be stocked in the department and used on demand. Though not yet commercially available, generic scalp bolus deserves further study.

Despite the recent advancements in TSI from bolus design to treatment techniques, there is variability in clinical practice. This current study aims to develop a streamlined workflow for the bolus design and VMAT plan optimization. Bolus fabrication time and adherence to the scalp are evaluated in a phantom study for three types of silicone bolus: flat, hat, and custom. VMAT optimization objectives are evaluated for planning target volume (PTV) coverage and OAR sparing. More specifically, the VMAT optimization technique is based on generalized equivalent uniform dose‐based (gEUD) objectives. The dosimetric effects of air gaps for the different types of boluses are also analyzed for plans optimized using the gEUD settings that best achieve the clinical goals.

## 2 METHODS

**FIGURE 1:**
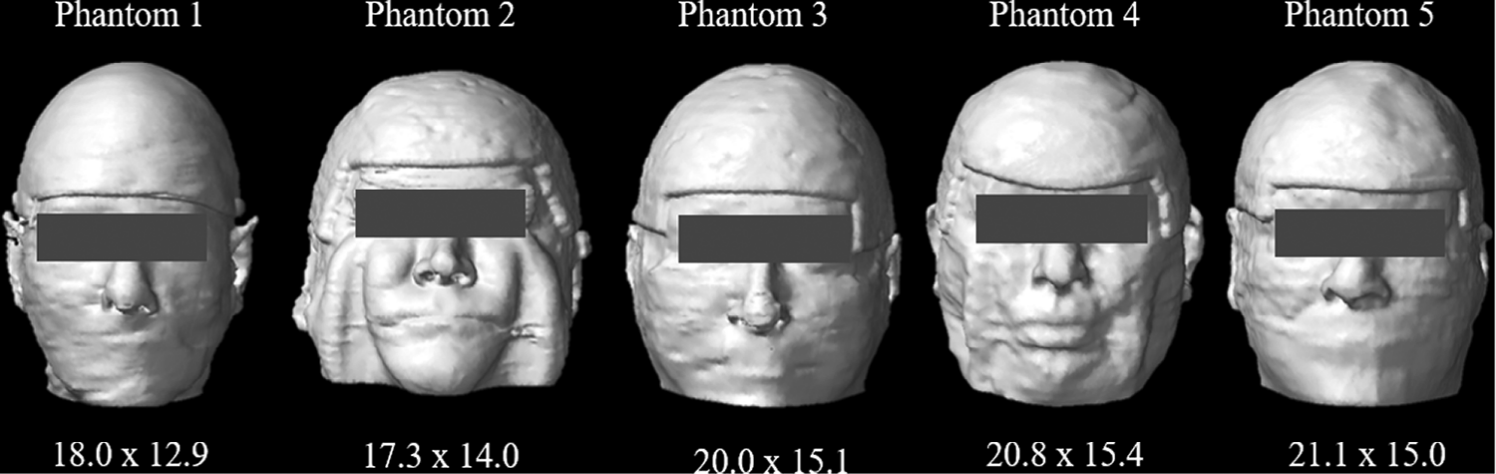
Renderings of the five 3D printed head phantoms with flat bolus in place. Head measurements above the brow are reported for the anterior‐posterior and lateral dimensions [cm × cm].

With IRB approval under the umbrella of Retrospective Quantitative Imaging Analysis for the Improvement of Quality and Safety of Radiotherapy (IRB# 114349), a retrospective analysis was performed using five total scalp irradiation cases treated within the last 10 years at a large academic institution. All patient cases were anonymized. Adult head sizes spanned various shapes and sizes (Figure 1). The clinical bolus was segmented out of the patient CT images prior to printing the head phantoms. A radiation oncologist wired each head phantom to designate the treatment area, as is performed clinically for TSI patients. CT images were exported to the Varian Eclipse Treatment Planning System (TPS) Version 16.1 for air gap analysis.

A CT scan was performed for each head phantom without any bolus then again with each physical bolus in place. In total, there were four CT scans for each case. All of the bolus designs were 5 mm thick and were provided by .demical. The flat bolus type was designed in‐house from 30 cm × 30 cm sheets of silicone. An experienced therapist cut the flat bolus and pieced together multiple strips to form a bolus helmet covering the scalp, following a workflow that is used in clinical practice for TSI.

The hat bolus has the appearance of an elongated swim cap. For the hat boluses, we selected a set of five patients that represented the typical range of head sizes seen in our practice. Together with .decimal, measurements of each patient's head were taken from CT scans and a generic elliptical dome shape, or hat, was developed for three sizes (small, medium and large). These generic hats were produced for testing as ready‐to‐use scalp bolus. The appropriate size was determined by placing each size hat bolus on an individual head phantom and selecting the bolus that fit most snugly. This novel generic hat bolus is not in the vendor catalog and has not been studied yet for total scalp irradiation.

Custom scalp boluses were generated from the anonymized CT images using the .decimal tool that generates uniformly thick bolus from the body contour surrounding the scalp. The bolus was then exported as a contoured structure in a DICOM‐RT structure set file. The vendor supplied a software product that generated a full 3D model of the imported structure for fabrication. The bolus was then fabricated by injecting silicone between a 3D‐printed mold of the head phantom and a matching shell separated by 5 mm.

**FIGURE 2:**
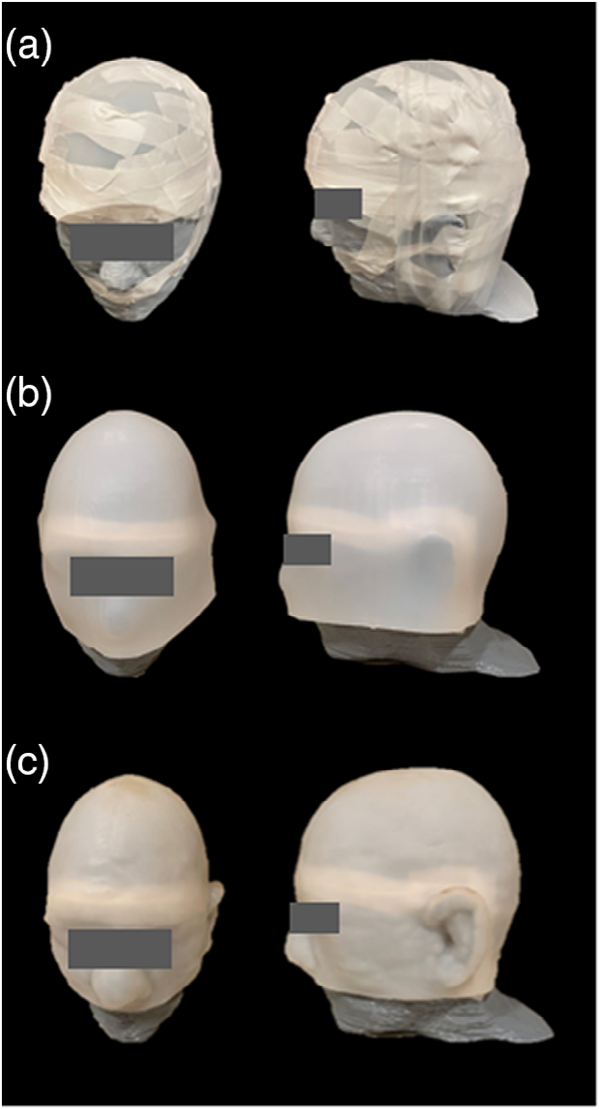
Frontal and lateral views of a 3D printed head phantom with 5 mm thick flat (a), hat (b), and custom bolus (c).

The three types of bolus are shown for a representative case in Figure 2. The time was recorded to modify the bolus, if needed, and place the bolus over the scalp. A wig cap was placed over each bolus per the vendor recommendations to improve adherence of the bolus to the scalp. A thermoplastic mask was then placed over the bolus before each CT scan to simulate patient immobilization for TSI.

**FIGURE 3:**
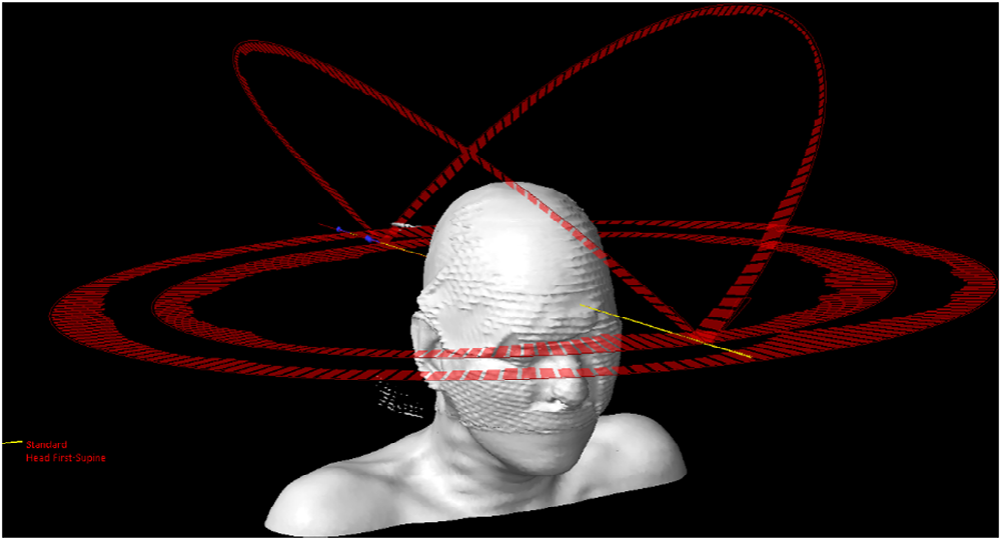
Beam configuration for TSI patients. Collimator jaws were set using the Arc Geometry Tool in Eclipse.

In parallel with the bolus analysis, VMAT planning techniques were studied using the anonymized patient datasets. This study used the original physician contours and the 5 mm thick clinical bolus, which was positioned on the patient at CT simulation. The plan isocenter was positioned in the brain at the PTV centroid. Each VMAT plan consisted of four arcs: two full circular arcs at couch 0° with the collimator at ±10° and two half‐circular arcs at couch ±45° with the collimator at 90°. Half arcs spanned from gantry head up to head down over the scalp superiorly, as shown in Figure  3. The collimator jaws were set to cover a PTV using the Arc Geometry Tool. Beam energy was 6 MV on a TrueBeam linac equipped with a Millennium MLC.

The optimization framework for sparing the brain and other healthy tissues was based on gEUD penalties. gEUD penalties were placed on two structures: the brain and an optimization structure including all tissues >8 mm from the PTV. The rationale for penalizing these structures is to allow tangential beams to enter and exit near PTV edges while penalizing those beamlets passing into the brain. For both structures, the gEUD dose was set to 4 Gy and the priority was fixed at 125. Then the gEUD alpha term was varied across the full range of values (0.1, 0.5, 1, 2, 3, 4, 5, 10, 20, and 40). Additionally, two objectives were placed on the PTV both with priorities of 250. The lower objective was 62.4 Gy, and the upper objective was 64.8 Gy. Optimization was performed using the Eclipse Photon Optimizer V16.1.0 with convergence mode set to off, on, and extended. Jaw tracking was enabled. Beam entry was avoided through the eyes. Following the dose calculation using the anisotropic analytical algorithm (AAA) on a 2.5 mm grid, the prescription dose of 60 Gy was set to cover 95% of a PTV. Brain and PTV doses were then analyzed across gEUD alpha values.

Using the optimal gEUD alpha value, the dosimetric effects of air gaps were studied on the phantoms for each type of bolus. For the air gap analysis, the PTV consisted of a shell within the region wired by the radiation oncologist that extended 5 mm below the surface of a head phantom. Note that the clinical bolus was segmented out prior to printing the head phantom, which is largely hollow. In the Eclipse treatment planning system, the phantom was overridden as water to mimic the brain. Then 5 mm thick virtual bolus was placed over the PTV. A VMAT TSI plan, as described above, was optimized for the ideal scenario of uniform bolus without any air gaps. This plan was then recalculated using prefixed monitor units on the CT scan of each physical bolus. The physical bolus and associated air gaps were depicted by CT values while the head phantom was again overridden as water. PTV coverage was evaluated at V57Gy and V60Gy. One‐way ANOVA and the Student's *t*‐test were used to assess for any statistically significant differences.

## 3 RESULTS

**FIGURE 4:**
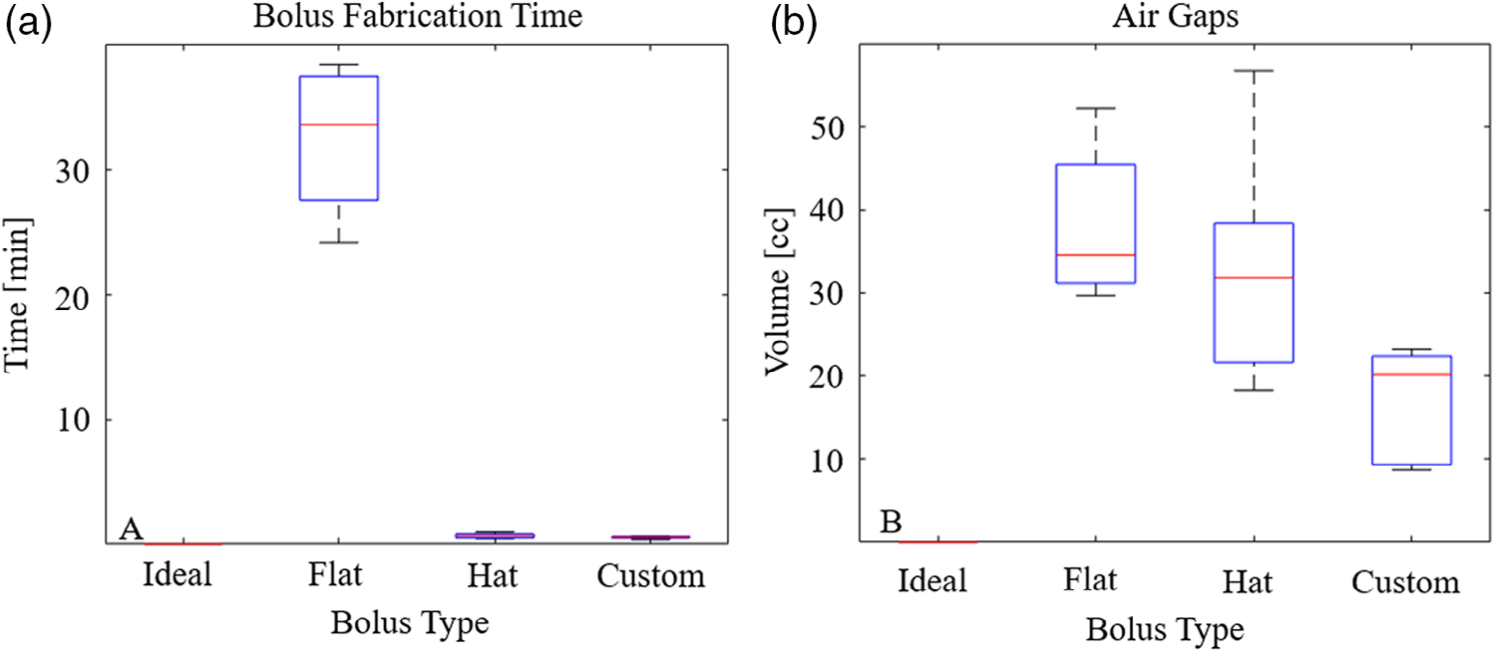
(a) (left) box plots of fabrication time at CT simulation for the silicone boluses (flat, hat and custom) on a log scale. (b) (right) air gap volumes for these respective boluses. The ideal, virtual bolus as modeled in Eclipse has no air gaps.

As shown in Figure 4(a), the time required to craft a scalp bolus from a flat silicone sheet and position it was 32 ± 5.9 min, compared with 0.67 ± 0.2 min for the generic hat bolus and 0.53 ± 0.10 min for the custom bolus (*p* < 0.001, one‐way ANOVA). Subsequent pair‐wise *t*‐test analysis showed non‐significant differences (*p* = 0.25) between the times to place the generic hat and custom boluses. However, the time to place either of these boluses was significantly shorter (*p* < 0.001) than the time to fabricate and place the flat sheet bolus.

**FIGURE 5:**
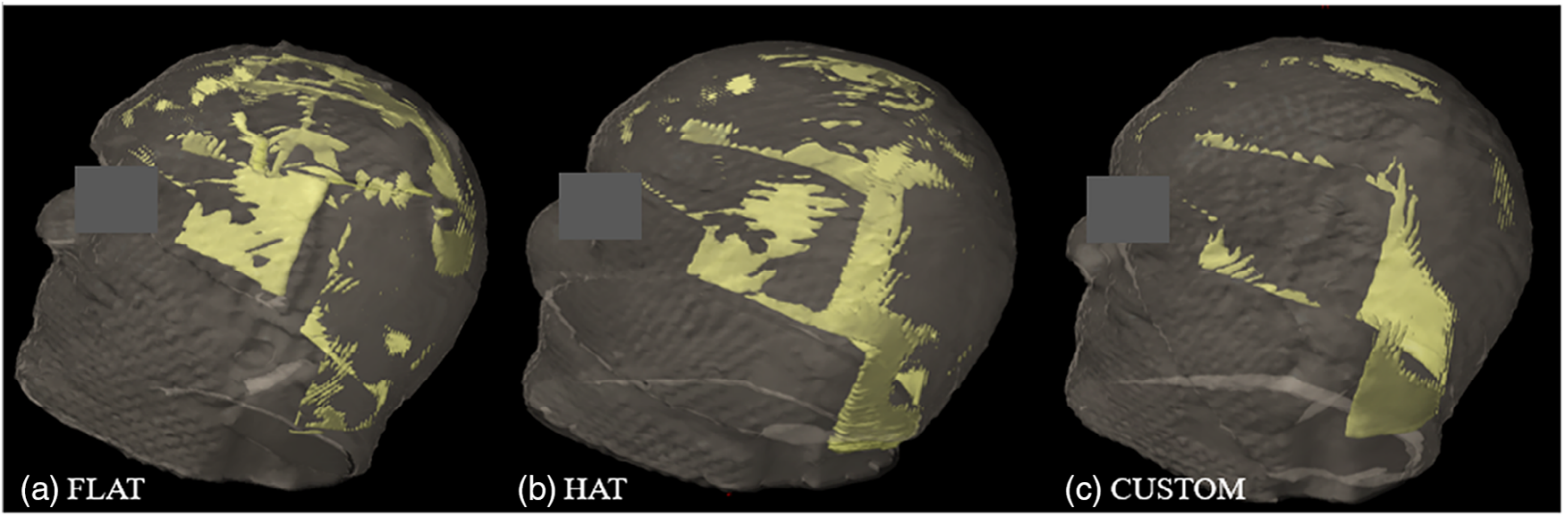
3D renderings of the body contour (gray) and air gaps (yellow) for (a) flat bolus cut to shape, (b) generic hat bolus, and (c) custom bolus crafted from a 3D printed mold of the body contour.

**FIGURE 6:**
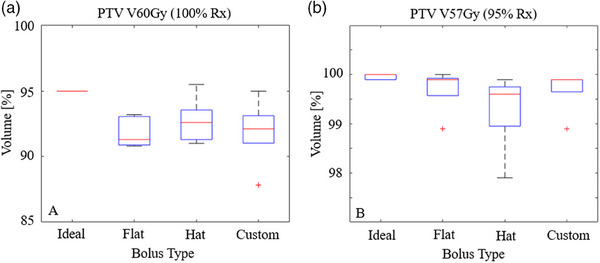
PTV dose coverage in the presence of air gaps between the bolus and scalp. (a) (left) shows the percentage of a PTV covered by the prescription dose, which was set at 95% for the plan with ideal bolus. (b) (right) shows the percentage of the PTV covered by 95% of the prescription dose (57 Gy); for all bolus types, V57Gy exceeds 97.5%.

Air gaps were observed under each physical bolus. Air gaps tended to be more pronounced behind the ears and laterally on the neck where contact is not made with the bolus by the thermoplastic mask or headrest, as shown in Figure 5. This effect is seen even with a wig cap placed over the bolus. The magnitude of the air gaps did vary with the bolus type. Across the five head phantoms, the air gaps volumes were 38 ± 9.3 cc for the flat bolus, 32 ± 14 cc for the generic hat bolus, and 17 ± 7.0 cc for the custom bolus (*p* = 0.024) as shown in Figure 4(b). Subsequent pair‐wise *t*‐test analysis showed a statistically significant difference (*p* = 0.011) between the flat and custom boluses; other differences were not significant. The effect of bolus type on target coverage (V60Gy and V57Gy), shown in Figure 6, was also evaluated using one‐way ANOVA (*p* = 0.75 and 0.45, respectively). Target coverage was similar for the different bolus types at 95% and 100% of the prescription dose.

**FIGURE 7:**
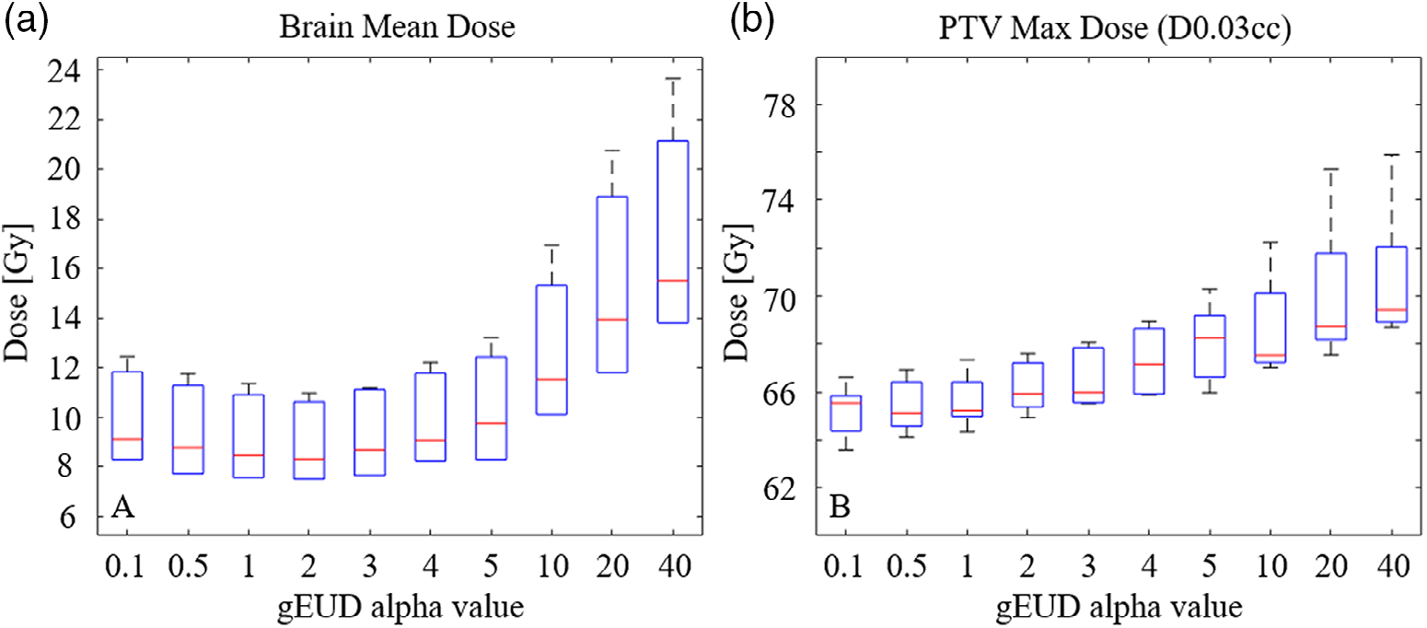
(a) (left) Box plots of mean brain dose as a function of gEUD alpha value across the five cases. Alpha = 2 results in the lowest mean brain dose, which had a clinically acceptable PTV D0.03 cc in the range of 64–68 Gy as shown in (b) (right).

**FIGURE 8:**
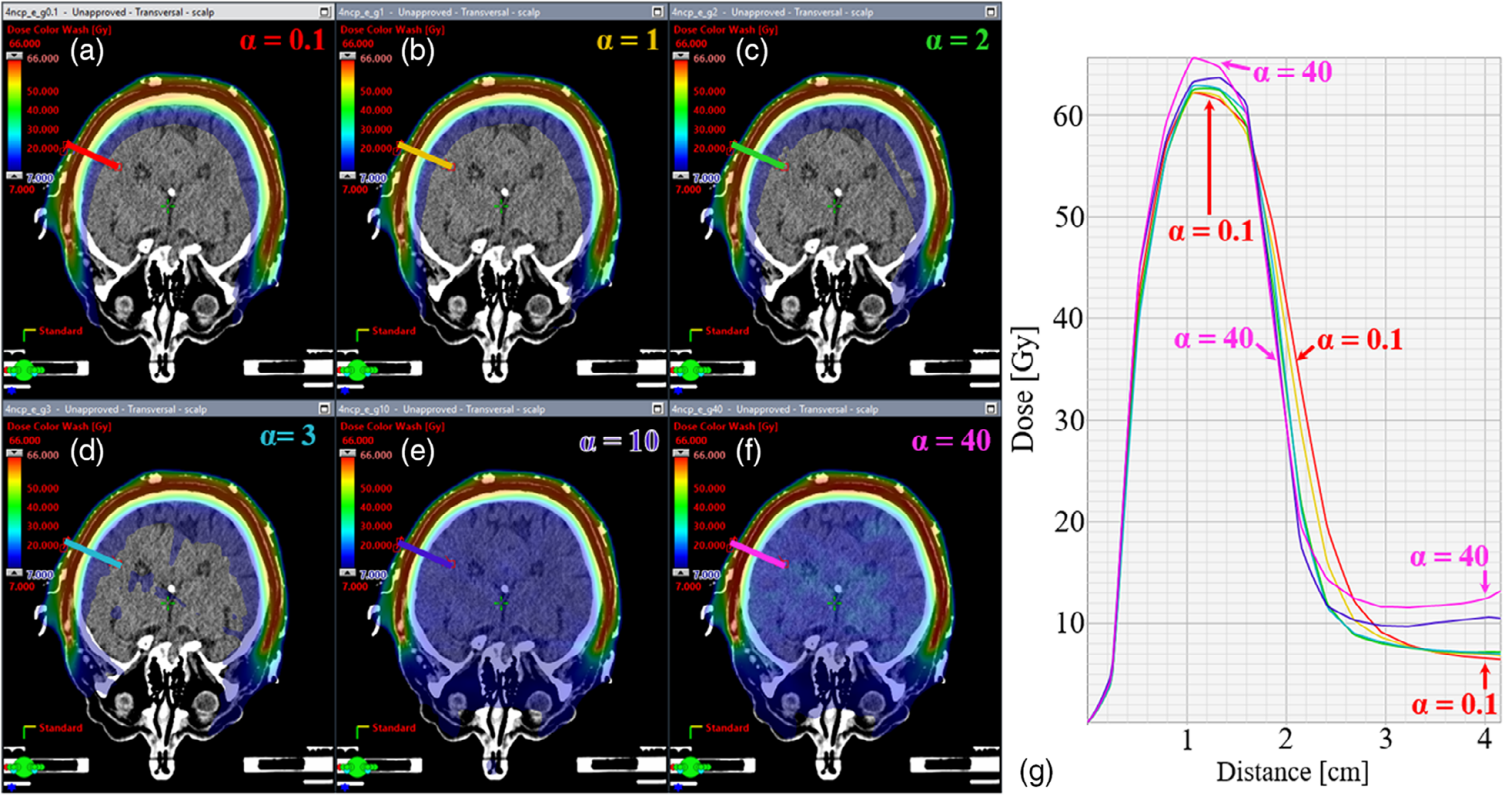
Representative dose distribution (a–f) and associated dose profile (G) across the full range of gEUD alpha values: (a) 0.1, (b) 1, (c) 2, (d) 3, (e) 10, and (f) 40. The lowest alpha value (0.1) achieves the best PTV dose uniformity and preferentially reduces the dose mid‐brain but with the tradeoff of a shallower dose gradient from the PTV to the brain. In contrast, the highest alpha value (40) has the steepest initial dose gradient but with the tradeoffs of a higher dose mid‐brain and a higher max dose within the PTV.

**FIGURE 9:**
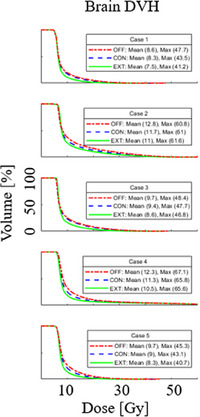
Brain dose volume histograms (DVHs) for the five cases with convergence mode OFF (red dashed dot curve), CONvergence on (blue dashed curved), and EXTended convergence mode (green solid curve). In each case, brain sparing was best using extended convergence mode.

The VMAT optimization study showed that across the gEUD alpha values of 0.1–40, an alpha of 2 resulted in the lowest mean brain dose (Figure 7a). Mean brain dose was slightly higher at the lowest alpha setting of 0.1 and notably higher at the highest alpha setting of 40. As alpha increased, so did the maximum dose within the PTV (Figure 7b). For a representative case, the dose distributions and dose profiles are shown for a range of alpha values (Figure 8). This figure gives spatial context to Figure 7. For a low alpha, PTV dose is uniform; however, the dose gradient into the brain is relatively shallow before plateauing at 10% of the prescription dose. In contrast for the highest alpha, PTV dose is less uniform while the dose gradient into the brain is the steepest. The dose midbrain, however, is around 20% of the prescription. An alpha of 2 balances the initial gradient and midbrain dose to achieve the lowest mean brain dose. Extended convergence mode resulted in better brain sparing as compared to the convergence mode settings of on or off, as shown in Figure 9. The rank order of brain sparing was best with extended convergence, followed by convergence mode on, and lastly with convergence mode turned off during optimization.

## 4 DISCUSSION

The VMAT optimization component of this study evaluated the impact of the gEUD alpha values on brain dose (Figures 6a and 7). The gEUD objective was applied to the brain, and an optimization structure included all healthy tissues > 8 mm from the PTV boundary. This optimization structure included radiosensitive OARs in the head and neck region while allowing for a margin along the PTV edge where tangential beams can enter and exit without penalizing the small dose extrusions. This approach allows for a non‐uniform gradient with greater importance placed on steepening the dose gradient between the PTV and brain. The choice of the gEUD alpha value was found to be important. A high alpha value prioritizes reduction of the high dose region, translating to the steep dose gradient between the PTV and brain seen in this study. A low alpha value places greater importance on the low dose region, while an alpha of one is equivalent to a mean dose objective. An alpha value of 2 was found to minimize the mean brain dose better than other alpha values. Using alpha of 2, the dose dropped from 60 Gy at the PTV edge to approximately 6 Gy at 2 cm away in the brain. A poorly selected alpha value resulted in a 10–15 Gy dose increase in the middle of the brain—dose levels that could cause cognitive decline. Extended convergence mode showed the best brain sparing and typically produced the most homogenous PTV coverage, albeit with the tradeoff of longer optimization time. These dose differences may translate to changes in cognitive performance for TSI patients, as has been observed in patients receiving hippocampal‐sparing whole‐brain radiation therapy. The final dose calculations were conducted using AAA with inhomogeneity corrections turned on, which represent the default settings within our institution. An additional investigation is needed to assess the accuracy of various dose calculation algorithms employed using this treatment planning technique, aiming to establish a recommended approach for clinical application. From a planner's perspective, the optimization framework used in this study was relatively fast and straightforward to implement, meaning that it may be readily translated in routine clinical practice.

Bolus fabrication can be a time‐consuming part of patient simulation for TSI. Results from this study show that around 30 min are needed to cut and piece together flat bolus into a helmet covering the scalp. When using flat sheets of silicone, silicone tape is superior to silk tape for securing multiple pieces of bolus together. In contrast, the alternative generic hat and custom boluses can be positioned in under 1 min. This time difference could meaningfully impact the workflow of a busy clinic. We also evaluated the quality of different bolus types by quantifying air gaps between the bolus and scalp and their dosimetric impact on target coverage. Bolus crafted from the flat sheet resulted in the most significant air gaps, despite the involvement of a seasoned therapist with expertise in these cases. While air gaps were reduced using a generic hat bolus, the difference was not statistically significant as compared with the flat bolus. Air gaps were the smallest using the custom bolus; this difference was significant compared to the flat bolus. However, the differences in air gap volumes did not translate to any meaningful changes in PTV coverage at V60Gy or V57Gy. Each bolus was similarly effective at increasing the superficial dose.

Based on these results, both the generic hat and custom bolus offers significant time saving while achieving similar target coverage compared to an in‐house bolus crafted from a flat sheet of silicone. The generic hat bolus has the advantage that it can be stocked and used on demand as needed in the clinic. In contrast, the custom bolus requires an upfront CT image of the patient followed by a modest turnaround time of a few days for bolus fabrication and shipping. The advantage of the custom bolus is that it can conform to patient‐specific anatomies, such as the ears, brow, or nose, which may account for the slightly shorter placement times compared to the generic cap bolus. It is important to note that the cases investigated in this study had relatively smooth scalps without significant defects from surgery or bulky disease that are often present in advanced skin cancers. Therefore, caution should be used if extrapolating these findings to other bolus applications over an irregular surface.

Bolus adherence was studied on plastic phantoms. A limitation of the current study is that the generic scalp bolus covered the eyes, nose, and mouth. In clinical practice, bolus would be cut away from this region; however, due to a limited number of bolus samples, the hat bolus was left intact. Further, the phantoms used in this study were hard plastic with a slightly rough surface attributable to the limited spatial resolution of the CT images and 3D printer. The spatial resolution was primarily limited by the CT slice thickness of 0.6 mm as compared to the 0.2 mm layer spacing of the 3D printed silicone molds. Likewise, the inplane CT voxel widths 1.0 mm are also larger than the in‐plane 3D printed mold resolution of 0.05 mm. While the plastic phantoms were anthropomorphic and spanned a range of head shapes and sizes, future studies are needed with actual patients to consider other essential aspects such as comfort, bolus reproducibility, and integrity over an extended period representative of the treatment course. The robustness of daily setup variations has yet to be studied in terms of air gap variations and the impact on the dose distribution. At the time of this phantom study, our clinical workflow relied on crafting bolus from flat sheets. However, the encouraging results from the generic hat and custom boluses provide impetus to consider a change in clinical practice.

## 5 CONCLUSION

This study investigates a VMAT planning technique and three types of bolus to improve the efficiency and plan quality of TSI. A straightforward optimization technique using well‐tuned gEUD objectives achieved the clinical goals of target coverage and brain sparing. Two types of silicone bolus—generic hat and patient‐specific custom bolus—resulted in significant time savings at simulation compared to cutting and piecing together strips of the flat bolus. The results demonstrate the potential to improve efficiency while achieving high plan quality.

## AUTHOR CONTRIBUTION

**Tanisha Davis**: Investigation; Writing—original—draft; Visualization. **Kirk Luca**: Investigation; Writing—original—draft; Writing—Review & editing; Supervision. **Lisa J. Sudmeier**: Writing—review & editing. **Zachary S. Buchwald**: Writing—review & editing. **Mohammad K. Khan**: Writing—review & editing. **Xiaofeng Yang**: Writing—review & editing. **Eduard Schreibmann**: Writing—review & editing. **Jiahan Zhang**: Conceptualization; Methodology; Formal analysis; Data curation; Writing—review & editing; Funding acquisition. **Justin Roper**: Conceptualization; Methodology; Formal analysis; Investigation; Data curation; Writing—review & editing; Visualization; Supervision; Project administration.

## CONFLICT OF INTEREST STATEMENT

The authors declare no conflicts of interest.

## Data Availability

Research data are stored in an institutional repository and will be shared upon request to the corresponding author.
